# In vivo changes in plasma acute phase protein levels in the rat induced by slow release of IL-1, IL-6 and TNF

**DOI:** 10.1155/S0962935192000085

**Published:** 1992

**Authors:** E. J. Lewis, A. D. Sedgwick, T. H. P. Hanahoe

**Affiliations:** 1 Biology Department Roche Products Ltd Broadwater Road Welwyn Garden City Herts AL7 3AY UK; 2 Assistant Director Hatfield Polytechnic College Lane Hatfield, Herts UK

## Abstract

Administration of large doses of cytokines by injection is required to induce changes in acute phase protein levels. Comparisons were made in the rat of the effects of administering recombinant human cytokines by injection with continuous release from implanted osmotic minipumps. Continuous release of interleukin-1β (0.2–2.1 ng h^-1^) induced dose-related changes in the plasma levels of albumin, seromucoid proteins, haptoglobin and caeruloplasmin; interleukin-1α had similar effects but required higher doses (2–21 ng h^-1^). Tumour necrosis factor α (50 ng h^-1^) only significantly increased seromucoid levels, whereas IL-6 (3–30 ng h^-1^) induced haptoglobin and caeruloplassynthesis. This method provides a better technique for studying the in rive effects of cytokines which may be relevant to the release mechanisms in inflammation.


Mediators of Inflammation 1, 39–44 (1992)

**ADMINISTRATION of large doses of cytokines by injection is required to induce changes in acute phase protein levels. Comparisons were made in the rat of the effects of administering recombinant human cytokines by injection with continuous release from implanted osmotic minipumps. Continuous release of interleukin-1$\beta$ (0.2–2.1 ng $h^{-1}$) induced dose-related changes in the plasma levels of albumin, seromucoid proteins, haptoglobin and caeruloplasmin; interleukin-1$\alpha$ had similar effects but required higher doses (2–21 ng $h^{-1}$). Tumour necrosis factor $\alpha$ (50 ng $h^{-1}$) only significantly increased seromucoid levels, whereas IL-6 (3–30 ng $h^{-1}$) induced haptoglobin and caeruloplas- synthesis. This method provides a better technique for studying the *in vivo* effects of cytokines which may be relevant to the release mechanisms in inflammation.**

**Key words:** Acute phase proteins, Interleukin-1, Interleukin-6, Tumour necrosis factor

### *In vivo* changes in plasma acute phase protein levels in the rat induced by slow release of IL-1, IL-6 and TNF

**E. J. Lewis,[CA] A. D. Sedgwick and T. H. P. Hanahoe[1]**

Biology Department, Roche Products Ltd, Broadwater Road, Welwyn Garden City, Herts AL7 3AY;
[1]Assistant Director, Hatfield Polytechnic, College Lane, Hatfield, Herts, UK

[CA] Corresponding Author

### Introduction

Acute phase proteins (APPs) are defined as those proteins produced by the liver whose levels change during the course of inflammation or trauma.[1,2] APP are heterogeneous in nature with different physiological functions, protein synthesis and often electrophoretic mobility and cannot be classified under one simple chemical heading. APP include proteins such as C-reactive protein, fibrinogen, haptoglobin, caeruloplasmin, albumin etc. In man, during the course of severe inflammation, levels of CRP can increase up to 1 000-fold and fibrinogen two- to three-fold. However, albumin levels can decrease, thus specific APPs also have a variable degree of change.

The change in levels of APPs has been shown to correlate to the degree of inflammation in patients with rheumatoid arthritis[3] and for this reason, APPs are routinely used as markers of inflammation and the effectiveness of drugs modifying arthritis. In the early 1970s it was shown that a plasma-borne mediator released from cells at the site of inflammation altered the rate of liver synthesis of the acute phase proteins.[4] This mediator was later purified, defined as a cytokine and named interleukin-1. Subsequently, a number of cytokines involved in inducing changes in liver synthesis have been identified, purified and cloned. The main cytokines recognised as inducing changes in liver synthesis of APPs are interleukin-1 (IL-1), interleukin-6 (IL-6) and tumour necrosis factor (TNF). All of these cytokines have been shown to induce APP changes in cultured hepatocytes,[5] hepatoma cells[6,7] and also induce changes in the plasma levels of animals after injection.[8,9] The concentrations of the cytokines required to induce changes in APPs *in vivo* have been much greater than those used *in vitro* because of the short half-life of the cytokines in blood.[10,11]

In inflammatory conditions, release of the cytokines is more likely to be continuous rather than spasmodic, therefore to model these effects *in vivo* a method of sustained release of the cytokines is required. The aim of this study was to investigate the effects of interleukin-1$\alpha$ on APP levels in the rat after single and multiple injection and to compare these effects with IL-1$\alpha$ administered by slow release from implanted osmotic minipumps. Further studies were made using slow release of recombinant human IL-1$\beta$, TNF$\alpha$ and IL-6 in the rat in order to determine the role of these cytokines in the induction of individual APPs.

### Materials and Methods

*Animals:* In all the experiments described, female, Allen and Hanbury Hooded strain rats were used. The rats weighed between 90 and 160g and were aged between 8 and 12 weeks old. The animals were provided with tap water and Spillers PCD diet *ad libitum* throughout the experiment.

*Collection of plasma:* Ether anaesthetized rats were bled from the dorsal tail artery using a heparin rinsed (Liquemine, Roche; 500 U/ml) 1 ml syringe fitted with a 23G × 30 mm needle. Blood was collected and transferred to a 1.5 ml eppendorf tube and

© 1992 Rapid Communications of Oxford Ltd.

centrifuged for 5 min at 1500 × *g* in a MSE microcentrifuge. Plasma was either stored at 4°C if analysed within 3 days or frozen at −20°C to be analysed at a later date.

*Formulation of cytokines for administration:* The recombinant human cytokines were kind gifts from Dr P Lomedico, Hoffman La Roche, Nutley, New York (rhIL-1α and rhIL-1β); Dr Hooculi, Hoffman La Roche, Basel (rhTNFα) and Professor W Fiers, University of Ghent, Belgium (rhIL-6). In all experiments the concentrations of the cytokines required were achieved by diluting stock concentrations with a sterile saline solution containing 0.2% bovine serum albumin, which was used as a carrier protein for the cytokine. Stock solutions were kept frozen at −20°C and thawed immediately before use. The specific activity of the recombinant human cytokines were:- rhIL-1α — $2.1 \times 10^7$ U/mg; rhIL-1β — $2.4 \times 10^8$ U/mg; rhTNFα — $1.0 \times 10^8$ U/mg; and rhIL-6 — $3.1 \times 10^8$ U/mg as determined by the appropriate cellular assays.

*Administration of cytokines:* Injections of rhIL-1α were made using dilutions of the stock solutions in 0.2% bovine serum albumin and rats were injected with 2 ml kg of body weight$^{-1}$. Alzet osmotic minipumps (Model 2001; 1 μl h$^{-1}$) were filled in a sterile air flow cabinet with the cytokine. Before the pumps were implanted the rats were anaesthetized and their backs shaved with animal clippers. A small incision (10–15 mm) was made through the skin and a pair of blunt ended scissors was inserted through the incision on one side and the tissue connecting the skin layer to the muscle body wall layer was gently teased apart to form a channel of about 3 cm wide to a depth of 3 cm. An osmotic minipump was implanted through the incision to the end of the channel on one side of the backbone. The incision was then closed using two or three Michel clips (6 mm) and the area swabbed with 1% Hibitane® in 70% ethanol.

*Assay of plasma APP:* Plasma obtained from the rats was analysed using a Cobas-bio centrifugal analyser. The methods used to assay APPs have been previously described in detail[12] but, in brief, albumin levels were determined using the bromocresol green dye binding assay;[13] seromucoid by protein precipitation,[14] haptoglobin by the generation of peroxidase activity when bound to added methaemoglobin[15] and caeruloplasmin by its oxidase activity using *p*-phenylenediamine.[16]

### Results

*Effect of injected rhIL-1α:* Injections of rhIL-1α (6 ng g of animal body weight$^{-1}$) were made into groups of five rats using a variety of routes of administration. The animals were bled 24 h later. Haptoglobin levels were significantly increased by injected rhIL-1α and this was independent of the route of administration (Table 1). No effect of IL-1 administration was seen in plasma levels of seromucoid. Seromucoid is a crude precipitation fraction of glycosylated proteins which according to our two-dimensional immunoelectrophoretic studies is composed of $\alpha_1$ acid glycoprotein, $\alpha_1$ cysteine protease inhibitor and possibly hemopexin. Caeruloplasmin levels were unaffected whereas albumin plasma levels were significantly reduced in the groups of rats injected i.p. or i.v. but not s.c.

*Dose response to rhIL-1α administered s.c.* The effect of single s.c. doses of 2 and 6 ng g$^{-1}$ of IL-1α was compared to the effect of 2 ng g$^{-1}$ of IL-1α administered at 0, 2 and 4 h. Plasma was obtained 24 h after the initial injection. The multiple dose was far more effective than the single dose with greater changes in plasma levels of albumin, seromucoid, haptoglobin and caeruloplasmin in the multiple dosed rats (Table 2). Injection of IL-1α (6 ng g$^{-1}$ s.c.) in both experiments (Tables 1 and 2) induced different degrees of changes in haptoglobin

**Table 1**. Effect of injected rhIL-1α on plasma APP levels in the rat

| Substance administered | Dose (ng g$^{-1}$) | Route | Albumin (g l$^{-1}$) | Seromucoid (g l$^{-1}$) | Haptoglobin (g l$^{-1}$) | Caeruloplasmin (g l$^{-1}$) |
|---|---|---|---|---|---|---|
| Vehicle | — | | 29.3 ± 0.24 | 3.9 ± 0.27 | 0.21 ± 0.02 | 0.66 ± 0.01 |
| IL-1α | 6 | s.c. | 28.6 ± 0.44 | 4.4 ± 0.20 | 0.51 ± 0.01 *** | 0.69 ± 0.01 |
| IL-1α | 6 | i.p. | 28.3 ± 0.18 * | 4.5 ± 0.25 | 0.47 ± 0.01 *** | 0.68 ± 0.02 |
| IL-1α | 6 | i.v. | 27.7 ± 0.24 ** | 4.3 ± 0.15 | 0.44 ± 0.05 | 0.69 ± 0.02 *** |

Values represent the mean ± SE from groups of five rats. Significant changes in the levels of albumin (*$p < 0.05$, **$p < 0.01$) were obtained from rats injected i.p. and i.v. respectively. Plasma was taken 24 h after injection. Administration of IL-1α by the different routes induced significant changes in haptoglobin level (*** $p < 0.001$) compared to vehicle (0.2% albumin in saline i.p.) dosed rats by Student's *t*-test (unpaired, two-tailed). Other values shown were not statistically different from the vehicle dosed group.

**Table 2.** Effect of single and multiple injections of rhIL-1 on plasma APP levels in the rat

| Substance administered | Dose (ng $g^{-1}$) | Number of doses | Albumin (g $l^{-1}$) | Seromucoid (g $l^{-1}$) | Haptoglobin (g $l^{-1}$) | Caeruloplasmin (g $l^{-1}$) |
|---|---|---|---|---|---|---|
| Vehicle | | | 26.8 ± 0.36 | 2.9 ± 0.19 | 0.44 ± 0.09 | 0.67 ± 0.01 |
| IL-1α | 2 | 1 | 25.5 ± 0.82 | 2.6 ± 0.14 | 0.64 ± 0.05 | 0.67 ± 0.03 |
| IL-1α | 6 | 1 | 26.8 ± 0.57 | 3.1 ± 0.12 | 0.65 ± 0.04 | 0.72 ± 0.02 * |
| IL-1α | 2 | 3 | 26.0 ± 0.63 | 3.6 ± 0.27 * | 1.05 ± 0.06 *** | 0.80 ± 0.03 *** |

Values represent the mean ± SE from groups of five rats. Significant changes in the levels of caeruloplasmin (*$p < 0.05$) were obtained from rats injected s.c. at a single dose of 6 ng $g^{-1}$. Plasma was taken 24 h after injection. Administration of IL-1α by multiple dose of 2 ng $g^{-1}$ at 0.2 and 4 h induced significant changes in the levels of seromucoid (*$p < 0.05$), haptoglobin (***$p < 0.001$) and caeruloplasmin (***$p < 0.001$) compared to vehicle (0.2% albumin in saline s.c.) dosed rats by Student's *t*-test (unpaired, two-tailed). Other values shown were not statistically different from the vehicle dosed group.

and caeruloplasmin levels. The lack of significant effect of IL-1α on haptoglobin levels in the second experiment may be explained by the higher levels of haptoglobin in the vehicle dosed (control) animals and greater variation as defined by the standard error. Caeruloplasmin levels were statistically significantly raised in the second experiment but not the first where the change in levels was 7% and 5% of the control levels respectively, therefore the degree of change should be considered minor in comparison to inflammation induced changes of 80–100% over control levels.

*Osmotic minimump released rhIL-1α*: Alzet osmotic minipumps (Model 2001) were filled with either 0.2% albumin as a vehicle control or concentrations of rhIL-1α of up to 21 ng $\mu l^{-1}$. The pumps were implanted s.c. and allowed to release IL-1α for about 110 h (1 $\mu l\ h^{-1}$—top total dose 2.3 $\mu g$/rat). Animals were bled on the fourth day after implantation and the plasma analysed for APPs (Table 3). This time schedule and doses were chosen from preliminary experiments where it was shown that the maximum increase in APP levels occurred within 3 to 4 days after implantation and then the APP levels decreased, possibly due to tolerance to human IL-1. Implanting the minipump and cutting through the rat skin produced a minor inflammatory reaction which induced a significant increase in plasma levels of caeruloplasmin compared to untreated rats. Minipumps administering 0.2 ng $h^{-1}$ of rhIL-1α had no effect on plasma APP levels compared to animals with minipumps containing vehicle. Only albumin levels were reduced by 0.6 ng $h^{-1}$ of rhIL-1α, higher doses of 2.1, 6.3 and 21 ng $h^{-1}$ significantly altered the other APPs in a dose related manner. It was noticed that by day 4, a granuloma had formed around the higher dose rhIL-1α pumps with or without a collection of fluid/exudate. The amount of fluid

**Table 3.** Effect of osmotic minipump released rhIL-1 on plasma APP levels in the rat

| Substance administered | Dose (ng $h^{-1}$) | Albumin (g $l^{-1}$) | Seromucoid (g $l^{-1}$) | Haptoglobin (g $l^{-1}$) | Caeruloplasmin (g $l^{-1}$) |
|---|---|---|---|---|---|
| Vehicle | | 35.9 ± 0.89 | 5.6 ± 0.37 | 1.05 ± 0.04 | 0.76 ± 0.02 |
| No Minipump | | 38.2 ± 0.55 * | 5.6 ± 0.51 | 1.01 ± 0.07 | 0.68 ± 0.02 * |
| IL-1α | 0.2 | 35.1 ± 0.79 | 5.5 ± 0.28 | 1.07 ± 0.05 | 0.73 ± 0.02 |
| IL-1α | 0.6 | 33.4 ± 0.48 * | 5.0 ± 0.21 | 1.11 ± 0.03 | 0.80 ± 0.02 |
| IL-1α | 2.1 | 33.4 ± 0.27 ** | 6.6 ± 0.39 | 1.73 ± 0.14 *** | 0.88 ± 0.02 ** |
| IL-1α | 6.3 | 28.9 ± 0.39 *** | 9.3 ± 0.20 *** | 1.96 ± 0.10 *** | 0.93 ± 0.04 ** |
| IL-1α | 21 | 28.1 ± 0.27 *** | 11.5 ± 0.34 *** | 2.20 ± 0.04 *** | 1.11 ± 0.02 *** |

Values represent the mean ± SE from groups of five rats. Group designated as vehicle were implanted with Alzet osmotic minipumps containing 0.2% albumin in saline and releasing 1 $\mu l\ h^{-1}$. The APP levels in plasma were determined 4 days after implantation of the pumps. Rats dosed with vehicle alone from the minipumps has significantly lower plasma levels of albumin (*$p < 0.05$) and greater levels of caeruloplasmin (*$p < 0.05$) than non-implanted rats (no minimpump). Significant changes in the levels of albumin (*$p < 0.05$) were obtained from rats administered with 0.6 ng $h^{-1}$. Administration of IL-1α at higher doeses of 2.1, 6.3, and 21 n h induced dose dependent changes in all the APPs measured, most of the changes were statistically significantly different from the vehicle alone group (**$p < 0.01$ to ***$p < 0.001$) as determined by Student's *t*-test (unpaired, two-tailed). Other values shown were not statistically significantly different from the vehicle dosed group.

within the granuloma and the granuloma weight varied greatly between rats within each group, therefore the exact volume or weight could not be used as a parameter except for being present or absent.

*Dose response studies to cytokines released from minipumps:* The effects of recombinant human IL-1α, IL-1β, IL-6 and TNFα released from implanted osmotic minipumps on APP levels in the rat were studied in separate dose response experiments. The effect of the minipump released cytokine was compared to minipump released 0.2% albumin and expressed as a percentage. Implanted minipumps containing 0.2% albumin were used as a vehicle control because of the minor inflammatory effect of implanting the minipumps and because of the variation in base-line levels of APPs. The maximal % changes shown for IL-1α are consistent with the changes associated with (adjuvant) inflammation.[12]

Recombinant human interleukin-1β was the most potent cytokine tested with activity at 0.21 ng $h^{-1}$ where the cytokine significantly reduced levels of albumin. At doses of 2.1 ng $h^{-1}$ interleukin-1β induced changes in the plasma levels of albumin, seromucoid, haptoglobin and caeruloplasmin and

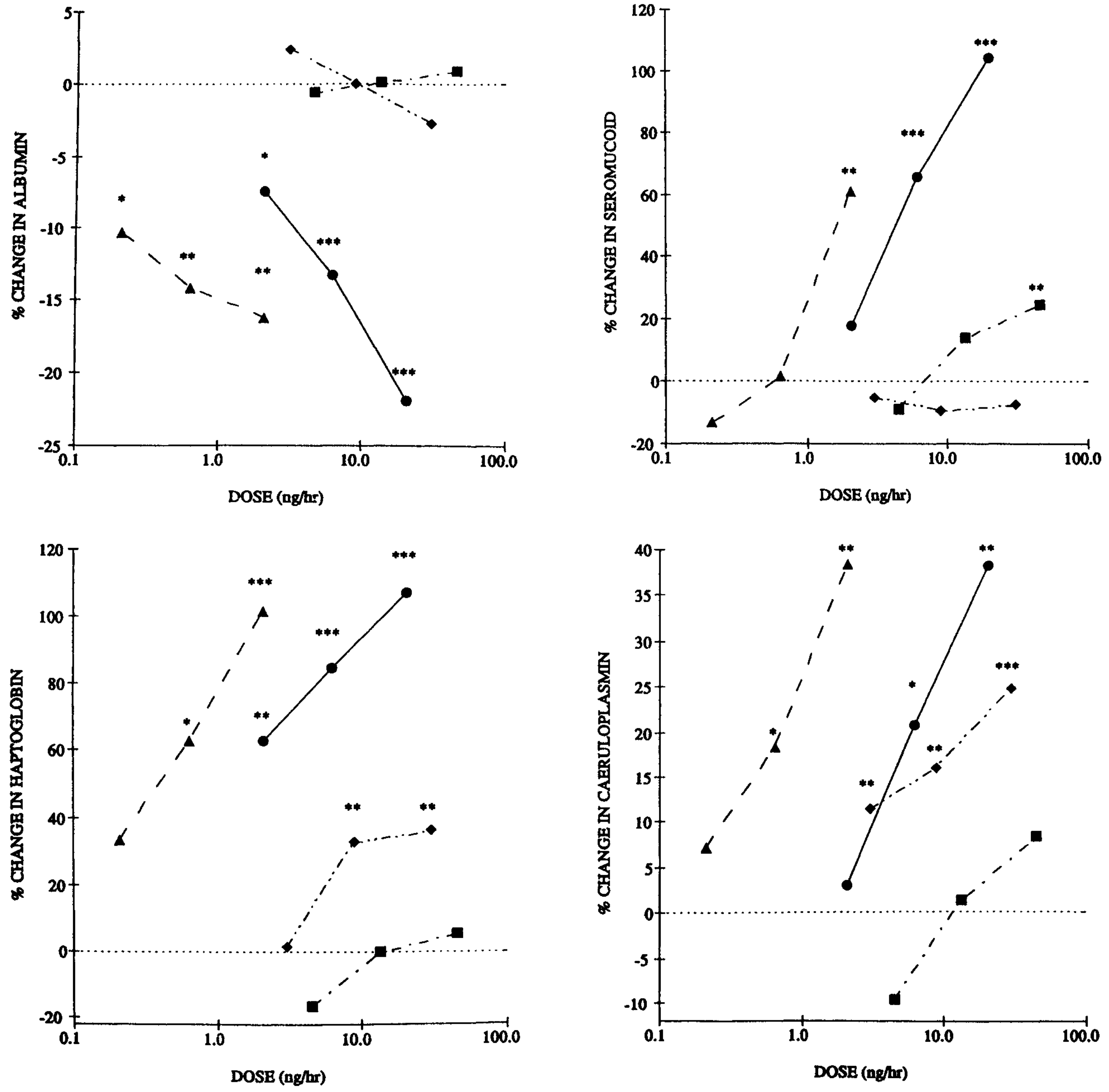

FIG. 1 Effect of osmotic minipump released cytokines on plasma APP in the rat. Figures show the change in individual APP levels in rats implanted with osmotic minipumps containing either cytokines or vehicle (0.2% albumin in saline). The dotted line shows the base level of the APP as 0% in the group of five rats implanted with osmotic minipumps containing the vehicle. All the effects of cytokine dosed rate (five rats/group) were corrected to the vehicle group and change in APP levels expressed as a % change. The effects of IL-1α (●–●); IL-1β (—▲—); IL-6 (–··–◆–··–) and TNFα (–·–■–·–) on plasma APP levels are shown after continuous release of the cytokines for 5 days. Statistical significance to the vehicle control group is shown as *$p < 0.05$; **$p < 0.01$; and ***$p < 0.001$ according to Student's $t$-test (unpaired; two-tailed).

also produced a variable exudate inside the granuloma in three out of five rats. Interleukin-1α had similar effects to those seen with IL-1β, but rhIL-1β was ten times more active than rhIL-1α in this rat model (Fig. 1).

At the highest dose tested (30 ng $h^{-1}$) rhIL-6 significantly increased haptoglobin and caeruloplasmin levels by 40% and 60% respectively when compared with the IL-1 induced levels. IL-6 did not produce granulomas or exudate fluid around the pump.

Recombinant human tumour necrosis factor α at doses up to 50 ng $h^{-1}$ did not induce granuloma formation or any exudate. The only change seen in the APP levels was a slight increase (20%) in the levels of seromucoid at the highest dose tested, 50 ng $h^{-1}$ (Fig. 1).

### Discussion

Administration of rhIL-1α by single injection with doses up to 600 ng/rat induced changes in the levels of APPs measured 24 h after injection, but these changes were not pronounced when compared with changes seen in inflammation. Administering rhIL-1α by multiple dose was more effective than the equivalent single dose with a significant change in levels of seromucoid, haptoglobin and caeruloplasmin. Similar results have been shown by De Jong *et al.*,[8] who demonstrated that i.p. injections of rhIL-1 at doses of 500 μg at 0, 2 and 4 h increased liver mRNA levels of $\alpha_1$ acid glycoprotein and transferrin but decreased albumin and fibrinogen. Also sustained short term release of rhIL-1 (150 μg i.v.) by a 6 h infusion in rats increased $\alpha_1$ acid glycoprotein but did not alter plasma levels of albumin, $\alpha_1$ cysteine protease inhibitor or $\alpha_2$ macroglobulin.[17]

Administration of IL-1α by sustained release from the osmotic minipump (6.3 ng $h^{-1}$) produced a greater response than a single injection (6 ng $g^{-1}$) even though the total dose administered was similar (approximately 600 ng/rat). Implantation of minipumps had only a minor effect on APP levels measured 4 days after implantation, therefore the enhanced activity of the cytokine must be dependent on the continuous release overcoming the rate of cytokine degradation. Few authors have used osmotic minipumps to administer cytokines when studying changes in APP levels; Gordeuk *et al.*,[18] have studied the effects of rhIL-1 on iron metabolism in mice and could not show any difference between single bolus and perfused IL-1 on the degree of decreased iron levels.[18] The dose related effects of rhIL-1α on APPs shows that the most sensitive APP of the number measured was albumin whose levels were decreased with administration of 0.6 ng $h^{-1}$. This sensitivity of the liver synthesis of albumin to low dose cytokines may have a beneficial effect *in vivo* by reducing albumin synthesis before allowing the liver to increase synthesis of other proteins.

Separate comparative studies using osmotic minipumps to administer IL-1α, IL-1β, IL-6 and TNFα were performed to determine which cytokine(s) modulated the synthesis of specific APPs in an *in vivo* model. In this model, implantation of the minipump caused a mild inflammation which induced a weak acute phase response (as seen with the increase in caeruloplasmin in the previous experiment), thus the levels of APP inducing cytokines and corticosteroids in the blood were probably raised above normal background levels. Changes in levels of the APPs were therefore compared to the APP levels from animals implanted with osmotic minipumps containing 0.2% albumin as a vehicle control.

Albumin levels were significantly reduced by both IL-1α and IL-1β but not IL-6 or TNFα. This effect of IL-1 is probably via a direct mediation because IL-1 can induce TNFα and IL-6 release from cells[19,20] which could alter liver APP synthesis. *In vitro* studies by Baumann *et al.*,[6] indicate that IL-1, TNFα and IL-6 act to decrease albumin production by hepatocytes, and this data is supported by Andus *et al.*[21] Administration of IL-1α by repeat i.p. doses *in vivo* reduced albumin synthesis;[8] IL-6 did not induce such a pronounced reduction.[9] Gresser *et al.*,[22] have demonstrated a weak reduction in albumin levels after single administration of rmTNF. Co-administration of dexamethasone with cytokines in human hepatoma cell cultures has been shown to reverse hypoalbuminia associated with cytokines,[6] therefore endogenous corticosteroids may act to inhibit IL-6 and TNF albumin lowering effects but not the effect of IL-1.

The difference in potency between IL-1α and IL-1β in the rat has been previously reported by Ferreira *et al.*,[23] who measured IL-1 induced algesia in rats and found that IL-1β was 100 times more potent than IL-1α. Most *in vitro* experiments do show similar activity for both types of IL-1, therefore the difference in effect of the two recombinant human IL-1's may relate to a metabolic effect or distribution of the cytokine in the rat.

Seromucoid levels were increased by both IL-1α, IL-1β and TNFα, although TNFα only caused a minor increase in seromucoid levels (20% of total response to IL-1). Seromucoid is a crude precipitation fraction of glycosylated proteins such as $\alpha_1$ acid glycoprotein, $\alpha_1$ cysteine proteinase inhibitor and hemopexin. Interleukin-1 has been shown to increase $\alpha_1$ acid glycoprotein levels both *in vitro*[24] and *in vivo*.[21] Interleukin-6 had no effect on seromucoid levels which differs from the findings of Markinovic *et al.*,[25] but is similar to those of Geiger *et al.*,[24] who measured liver mRNA levels of acute phase proteins after i.p. injection of 800 ng of IL-6 but this dose only increased fibrinogen to a similar extent to that induced by injection of turpentine. In the same study $\alpha_1$ acid glycoprotein and $\alpha_1$ cysteine protease inhibitor were only marginally increased by IL-6.[9]

Haptoglobin levels were raised by IL-1α, IL-1β and IL-6. *In vitro* studies using hepatocytes have shown that IL-6 acts in synergy with IL-1 and glucocorticoids to induce both haptoglobin mRNA and protein.[26] *In vivo* studies have shown that IL-6 and IL-1 both induce haptoglobin levels in the rat.[25,27]

Caerulosplasmin is not a widely studied APP because levels of this protein are only doubled in inflammatory conditions. In these experiments caeruloplasmin levels were increased by IL-1 and IL-6 administration with IL-6 inducing 60% of the change seen with IL-1, thus IL-6 may be the main inducer of caeruloplasmin.

Both IL-1α and IL-1β produced a granulomatous response around the minipump, a similar finding has been shown by Dunn *et al.*,[28] who studied the effects of slow release cytokines from implanted ethylene vinyl acetate sponges. The fluid surrounding the pumps contained large numbers of red blood cells and PMNs which is consistent with the reported effects of IL-1β in inducing a Shwartzman-like reaction in rabbit skin after intradermal injections.[29] The infiltration of PMNs into the skin has also been reported to occur in rabbit skin after i.d. injection of IL-1β and IL-1α at doses of 0.1 ng[30] and 2.5 ng/injection site respectively.[31] No granulomatous response was seen with implanted osmotic minipumps containing vehicle, IL-6 or TNFα.

Overall, the results suggest that *in vivo* in the rat where there would be continuous release of IL-1, IL-6, TNF and glucocorticoids at basal levels, IL-6 may be the major inducer of caeruloplasmin and act in synergy with IL-1 and glucocorticoids to induce haptoglobin. IL-1 is probably the main inducer of the seromucoid proteins synthesis and hypoalbuminia. It would appear that TNF has only a minor role in the production of the seromucoid proteins.

The use of osmotic minipumps to infuse cytokines into animals has shown that greater effects can be obtained with 'low' concentrations of cytokines and also that this method may be physiologically more relevant than single bolus dose since in inflammatory conditions cytokines would be released continuously over the period of the inflammation.

### References

1. Koj A. *Structure and Function of Plasma Proteins.* London: Plenum Press, 1975; 73–131.
2. Whicher JT, Dieppe PA. Acute phase proteins. *Clin Immunol Allerg* 1985; **5:** 425–446.
3. Thompson PW, Silman AJ, Kirwan JR, *et al.* Articular indices of joint inflammation in rheumatoid arthritis. *Arth Rheum* 1987; **30:** 618–623.
4. Kampschmidt RF, Fuller GM. The effects of leucocyte endogenous mediator on plasma fibrinogen and haptoglobin. *Proc Soc Exp Biol Med* 1974; **146:** 904–907.
5. Koj A, Kurdowska A, Magielska-Zero D, *et al.* Limited effects of recombinant human and murine interleukin-1 and tumour necrosis factor on production of acute phase proteins by cultured rat hepatocytes. *Biochem Int* 1987; **14:** 553–560.
6. Baumann H, Richards C, Gauldie J. Interaction among hepatocyte-stimulating factors, interleukin-1, and glucocorticoids for regulation of acute phase plasma proteins in human hepatoma [hepG2] cells. *J Immunol* 1987; **139:** 4122–4128.
7. Baumann H, Onarato V, Gauldie J, *et al.* Distinct sets of acute phase plasma proteins are stimulated by separate human hepatocyte-stimulating factors and monokines in rat hepatoma cells. *J Biol Chem* 1987; **262:** 9756–9767.
8. De Jong FA, Birch HE, Schreiber G. Effect of recombinant interleukin-1 on mRNA levels in rat liver. *Inflammation* 1988; **12:** 613–617.
9. Geiger T, Andus T, Klapproth T, *et al.* Induction of rat acute phase proteins by interleukin-6 *in vivo. Eur J Immunol* 1988; **18:** 717–721.
10. Kampschmidt RF, Jones T. Rate of clearance of interleukin-1 from the blood of normal and nephrectomized rats. *Proc Soc Exp Biol Med* 1985; **180:** 170–173.
11. Castell JV, Geiger T, Gross V *et al.* Plasma clearance, organ distribution and target cells of interleukin-6/hepatocyte-stimulating factor in the rat. *Eur J Biochem* 1988: **177:** 357–361.
12. Lewis EJ, Bishop J, Cashin CH. Automated quantification of rat plasma acute phase reactants in experimental inflammation. *J Pharm Methods* 1989; **21:** 183–194.
13. Dow D, Pinto PVC. Determination of serum albumin on the SMA-12/30 (hospital model) using bromo-cresol green. *Clin Chem* 1969; **15:** 1006–1008.
14. Sternburg M, Peyroux J, Engler R, *et al.* Orosomucoid, seromucoid and haptoglobin in serum during adjuvant arthritis in the rat. *Experentia* 1974; **30:** 193–195.
15. Owen JA, Better FC, Hoban J. A simple method for determination of serum haptoglobins. *J Clin Pathol* 1960; **13:** 163–164.
16. Sunderman FW, Nomoto S. Measurement of human serum caeruloplasmin by its *p*-phenylenediamine oxidase activity. *Clin Chem* 1970; **16:** 903–910.
17. Tocco-Bradley R, Moldawer LL, Jones CT, *et al.* The biological activity *in vivo* of recombinant murine interleukin-1 in the rat. *Proc Soc Expt Biol* 1986; **182:** 263–271.
18. Gordeuk VR, Prithviraj P, Dolinar T, *et al.* Interleukin-1 administration in mice produces hypoferremia despite neutropenia. *J Clin Invest* 1988; **82:** 1934–1938.
19. Dinarello CA, Cannon JG, Wolff SM, *et al.* Tumor necrosis factor (cachectin) is an endogenous pyrogen and induces production of interleukin-1. *J Exp Med* 1986; **163:** 1433–1450.
20. Shalaby MR, Waage A, Aarden L, *et al.* Endotoxin, tumor necrosis factor-α and interleukin-1 induce interleukin-6 production *in vivo. Clin Immun Immunopath* 1989; **53:** 488–498.
21. Klapproth J, Castell JV, Geiger T, *et al.* Fate and biological action of recombinant interleukin-1β in the rat *in vivo. Eur J Immunol* 1989; **19:** 1485–1490.
22. Gresser I, Delers F, Tran Quangs N, *et al.* Tumour necrosis factor induces acute phase proteins in rats. *J Biol Reg Haemostat Agents* 1987; **1:** 173–176.
23. Ferreira SH, Loenzetti BB, Bristow AF, *et al.* Interleukin-1β is a potent hyperalgesic agent antagonized by a tripeptide analogue. *Nature* 1988; **334:** 898–890.
24. Geiger T, Andus T, Klapproth T, *et al.* Induction of $\alpha_1$ acid glycoprotein by recombinant interleukin-1 in rat hepatoma cells. *J Biol Chem* 1988; **263:** 7141–7146.
25. Marinkovic S, Jahres GP, Wong GG, *et al.* IL-6 modulates the synthesis of a specific set of acute phase proteins *in vivo. J Immunol* 1989; **142:** 808–812.
26. Baumann H, Morella KK, Jahreis GP, *et al.* Distinct regulation of the interleukin-1 and interleukin-6 response elements of the rat haptoglobin gene in rat and human hepatoma cells. *Mol Cell Biol* 1990; **10:** 5967–5976.
27. Baumann H, Prowse KR, Marinkovic S, *et al.* Stimulation of hepatic acute phase response by cytokines and glucocorticoids. *Ann N Y Acad Sci* 1989; **557:** 280–295.
28. Dunn CJ, Hardee MM, Staite ND. Acute and chronic inflammatory responses to local administration of recombinant IL-1α, IL-1β, TNFα, IL-2 and IFNγ in mice. *Agents & Actions* 1989; **27:** 290–293.
29. Movat HZ, Burrowes CE, Cybulsky MI, *et al.* Acute inflammation and a Shwartzman-like reaction induced by interleukin-1 and tumor necrosis factor. *Am J Path* 1987; **129:** 463–476.
30. Martin S, Maruta K, Burkart V, *et al.* IL-1 and IFNγ increase vascular permeability. *Immunology* 1988; **64:** 301–305.
31. Watson ML, Lewis GP, Westwick J. Increased vascular permeability and polymorphonuclear leucocyte accumulation *in vivo* in response to recombinant cytokines and supernatant from cultures of the human synovial cells treated with interleukin-1. *Br J Pharmac* 1989; **70:** 307–312.

**Received 18 October 1991;
accepted in revised form 4 November 1991**
